# Supplementary material for: Unveiling Epigenetic Vulnerabilities in Triple-Negative Breast Cancer through 3D Organoid Drug Screening
Source: Pharmaceuticals (Basel). 2024 Feb 8;17(2):225. doi: 10.3390/ph17020225 (PMC10892330; doi:10.3390/ph17020225)
Supplement: Supplementary file 1 [file pharmaceuticals-17-00225-s001.zip › supplementary files/figureS2.pdf]

Figure S2

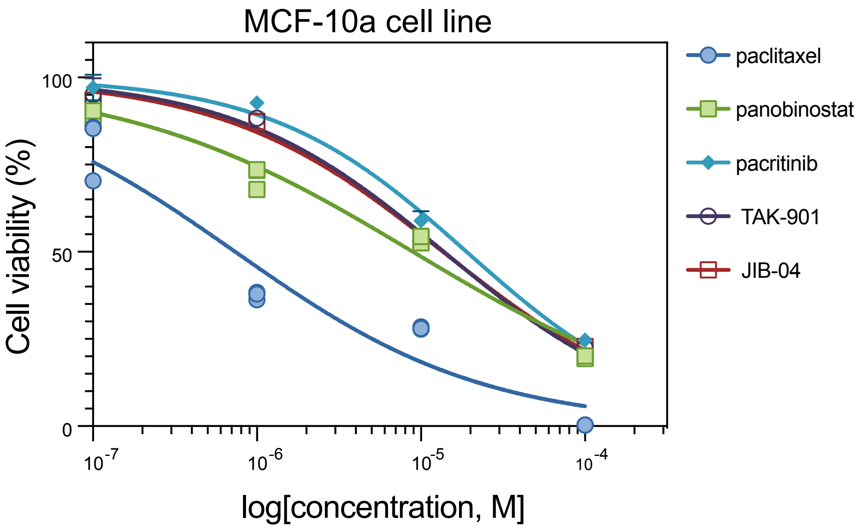

Figure S2. Dose-response curves for the non-tumorigenic epithelial MCF-10a cell line following treatment with paclitaxel, panobinostat, pacritinib, TAK-901, and JIB-04.
