# Supplementary material for: Unveiling Epigenetic Vulnerabilities in Triple-Negative Breast Cancer through 3D Organoid Drug Screening
Source: Pharmaceuticals (Basel). 2024 Feb 8;17(2):225. doi: 10.3390/ph17020225 (PMC10892330; doi:10.3390/ph17020225)
Supplement: Supplementary file 1 [file pharmaceuticals-17-00225-s001.zip › supplementary files/figureS1.pdf]

Figure S1

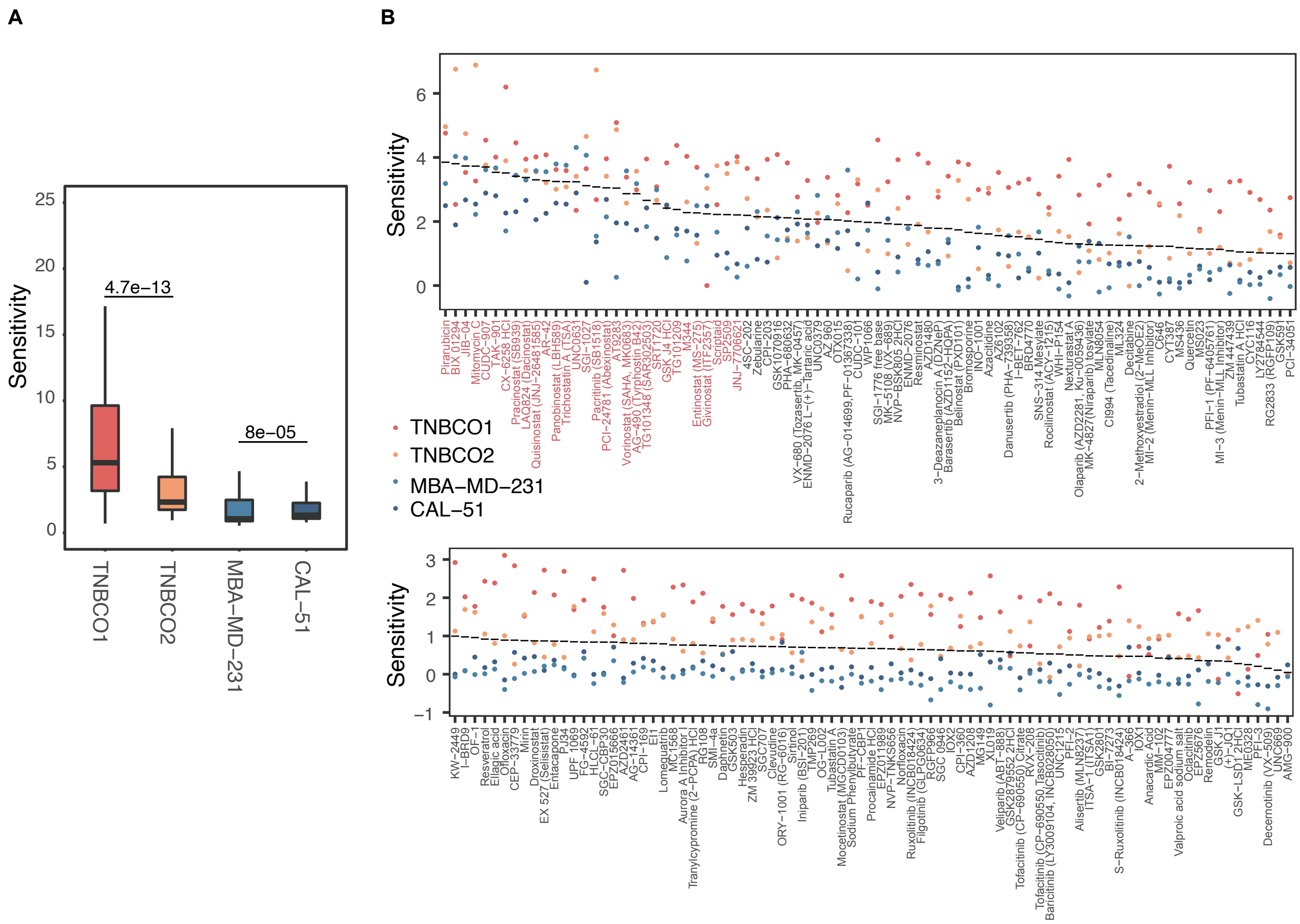

Figure S1. Preliminary screening reveals the remarkable precision of 3D organoid models compared to 2D cell lines in epigenetic drug selection. (A) Analysis of overall drug sensitivity in two TNBC organoids and two cell lines. (B) Scatter plot illustrating drug sensitivity, organized by the average sensitivity of two TNBC organoids and two cell lines for each drug. The top 30 drugs with the highest average sensitivity were chosen for secondary screening and are highlighted in red.
